# Role of prostaglandin E2 in tissue repair and regeneration

**DOI:** 10.7150/thno.63396

**Published:** 2021-08-13

**Authors:** Hui Cheng, Haoyan Huang, Zhikun Guo, Ying Chang, Zongjin Li

**Affiliations:** 1 Nankai University School of Medicine, Tianjin, China.; 2 The Key Laboratory of Bioactive Materials, Ministry of Education, Nankai University, the College of Life Sciences, Tianjin, China.; 3 Tianjin Key Laboratory of Human Development and Reproductive Regulation, Nankai University Affiliated Hospital of Obstetrics and Gynecology, Tianjin, China.; 4 Henan Key Laboratory of Medical Tissue Regeneration, Xinxiang Medical University, Xinxiang, China.; 5 State Key Laboratory of Kidney Diseases, Chinese PLA General Hospital, Beijing, China.

**Keywords:** Prostaglandin E2, Stem cell, Tissue repair, Regeneration, Inflammation

## Abstract

Tissue regeneration following injury from disease or medical treatment still represents a challenge in regeneration medicine. Prostaglandin E2 (PGE2), which involves diverse physiological processes via E-type prostanoid (EP) receptor family, favors the regeneration of various organ systems following injury for its capabilities such as activation of endogenous stem cells, immune regulation, and angiogenesis. Understanding how PGE2 modulates tissue regeneration and then exploring how to elevate the regenerative efficiency of PGE2 will provide key insights into the tissue repair and regeneration processes by PGE2. In this review, we summarized the application of PGE2 to guide the regeneration of different tissues, including skin, heart, liver, kidney, intestine, bone, skeletal muscle, and hematopoietic stem cell regeneration. Moreover, we introduced PGE2-based therapeutic strategies to accelerate the recovery of impaired tissue or organs, including 15-hydroxyprostaglandin dehydrogenase (15-PGDH) inhibitors boosting endogenous PGE2 levels and biomaterial scaffolds to control PGE2 release.

## Introduction

Effective tissue repair and regeneration after injury is critical for function maintenance and survival of all living organisms, which attract researchers to identify common regulators in tissue repair and regeneration 1. In recent years, increasing evidences have defined that prostaglandin E2 (PGE2), a lipid signaling molecule involved in pain and inflammation, might potentiate tissue regeneration and repair following injury in diverse organ systems 2-4. The utilization of nonsteroidal anti-inflammatory drugs (NSAIDs) reducing PGE2 levels in patients usually cause side effects on the tissue repair process 5-7. Thus, further understanding and exploring the universal and unique mechanism of PGE2 in the process of organ repair might contribute to the development of the field of regenerative medicine. Unexpectedly, PGE2 has a faster turnover rate *in vivo* due to degradation by 15-hydroxyprostaglandin dehydrogenase (15-PGDH), converting it into an inactivated biomolecule. In recent years, research on 15-PGDH inhibitors in the field of regeneration has gradually increased, while biomaterials delivery system delivery regulating PGE2 release has improved the regeneration efficacy of PGE2. Here, we summarize recent researches about PGE2 instructing tissue repair and regeneration in different organ systems including skin, heart, liver, kidney, gut, bone, skeletal muscle, and hematopoietic system. Then, we describe the advancement of therapeutic strategies for enhancing tissue regeneration with PGE2.

## The production and downstream signal of endogenous PGE2

PGE2, a mediator of many physiological and pathological functions 8, can be produced by nearly all cell types of the body, such as epithelia, fibroblasts, and especially infiltrating inflammatory cells. The production of PGE2 increases significantly in damaged tissue 9. Under diverse stimulation such as inflammation, PGE2 is generated from arachidonic acid (AA) that is liberated from membrane phospholipids catalyzed by phospholipase A2 (PLA2). Then the AAs are oxygenated to form Prostaglandin H2 (PGH2) by cyclooxygenase (COX), which is then transformed into PGE2 via terminal PGE2 synthases (PGES).

In detail, AA is immediately converted at the luminal side of nuclear and endoplasmic reticulum (ER)-membranes into the intermediate PGH2 by COX and converted into different prostaglandins by cell- and tissue-specific prostaglandin synthases (PGS). Constitutively expressed COX-1 is responsible for catalyzing the production of prostaglandins, which involve in several physiological functions. Inducible COX-2 is a rate-limiting enzyme for the synthesis of PGE2 and has been reported to be highly induced by inflammatory mediators following injury 9. Then, cytosolic prostaglandin E synthases (cPGES) and microsomal prostaglandin E synthases1/2 (mPGES-1/2) are responsible for the production of PGE2 from PGH2. mPGES-1 is mainly coupled with COX-2 to increase the production of PGE2, especially under the stimulation of inflammatory factors. However, mPGES-2 and cPGES expression are constitutive rather than regulatory. To perform further function, the synthesized PGE2 may be actively transported by the prostaglandin efflux transporter MRP4 (multidrug resistance protein 4) in addition to exit from the cell by simple diffusion 10. NSAIDs, widely used drugs in patients requiring pain reduction and inflammation control, not only decrease PGE2 production by suppressing COX activity but also inhibit MRP4-regulated PGE2 release 10, 11.

**Figure 1:**
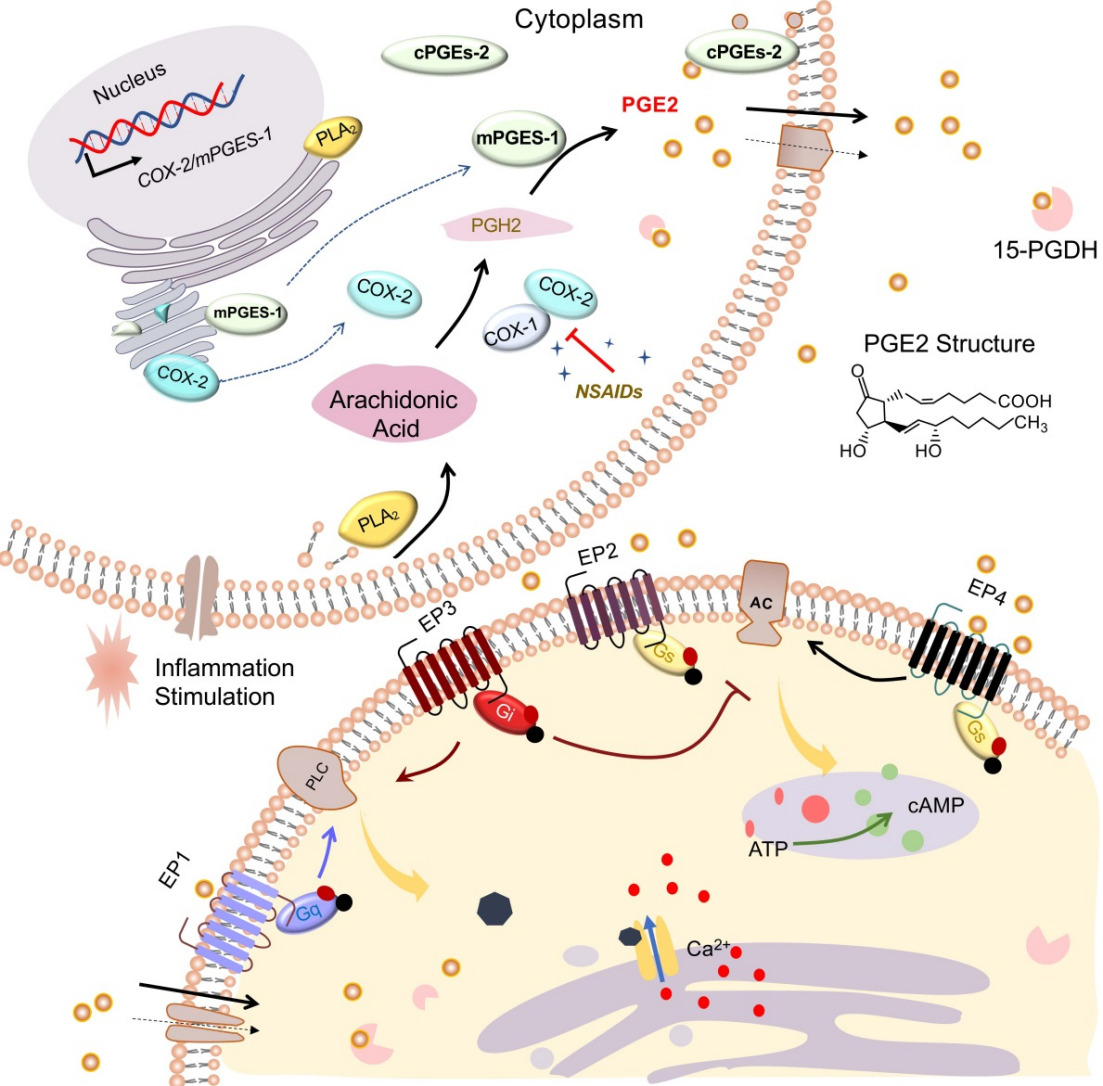
** PGE2 synthesis and signaling pathways.** Arachidonic acid (AA) is liberated from membrane phospholipids by catalysis of phospholipase A2 (PLA2), subsequently converted into PGH2 via COXs including COX-1 and COX-2. NSAIDs can restrain COX activity. Synthesized by PGE synthase (cPGES, mPGES-1, and mPGES-2), PGE2 exerts its effects by binding to G protein-coupled receptors EP1-EP4. Both activated EP1 (coupled to Gq) and EP3 (coupled with Gi) could increase intracellular Ca^2+^ via phospholipase C (PLC). Activated EP3 receptors could inhibit cAMP production via adenylate cyclase (AC). Activated EP2 or EP4 (both coupled to Gs) could elevate cAMP production via AC. Arrowheads refer to stimulation, whereas blunt ends indicate inhibition.

Subsequently, massively synthesized PGE2 will exert its diverse and complex biologic effects by binding to different downstream prostaglandin E receptors EP1, EP2, EP3, and EP4 that are located on the cell membrane or organelle membrane 12, 13. EP receptors belong to a large family of seven transmembrane domain receptors coupled to specific G proteins that activate different second messenger signaling pathways. The final output of PGE2 signaling depends on the expression of each EP receptor and the strength of each EP signal 14. EP1 (couple to Gq) and EP3 (couple to Gi) mediate PGE2-induced intracellular calcium mobilization. The EP2 and EP4 receptors coupled to Gs activate adenylate cyclase (AC) and increase cAMP production, whereas the EP3 receptor inhibits cAMP signaling. EP receptors, selective agonists, or antagonists were used to amplify or antagonize PGE2 signal effects 15-18. It's reported that solute carrier organic anion transporter family member 2A1 (SLCO2A1), known as prostaglandin transporter (PGT), is responsible for transporting extracellular PGE2 into the cell and performs a function in the intracellular activity of PGE2 13, 19. However, PGE2 influx mediated by SLCO2A1 is rapidly metabolized into inactivated 15-keto PGE2 by 15-PGDH 19, 20. Therefore, inhibition 15-PGDH may be an effective way to elevate PGE2 levels and enhance its downstream signal effects 21, 22. Finally, we summarize the overall process of PGE2 from synthesis to activating downstream signaling in **Figure 1**.

## The role of PGE2 on organ repair and regeneration

### PGE2 and cutaneous wound healing

Non-healing cutaneous wounds caused by surgery, diabetes, or extreme temperature, have always been a severe spirit and financial burden for patients with abnormal wound healing 23, 24. Normal wound healing processes comprise several overlapping phases including hemostasis, inflammatory response, proliferation, and tissue remodeling. Among them, the proliferation stage includes re-epithelialization, granulation tissue formation, and angiogenesis. If one or more stages of the wound healing process are interrupted or dysregulated, the healing rate will be delayed. In the cutaneous wound model, the expression of COX-2/PGE2/EP4 rises shortly after damage 25, 26. Elevated PGE2 level accelerates the cutaneous wound healing process 25, 27. Even though COX-1 is expressed constitutively, it still benefits PGE2 production and prevents wound healing disorders 28.

PGE2 displays great promise for the therapy of excisional skin wounds as it participates in different pathological repair processes with its function of anti-inflammatory, promoting angiogenesis, especially preventing scar formation 2. To effectively keep the concentration of PGE2, we incorporated PGE2 into chitosan hydrogel to treat injured sites in a murine model of cutaneous wound healing 2. Consistent with previous reports, PGE2 not only accelerates the healing rate but also remodels the skin structure in injured sites with new hair follicles and sebaceous glands. Furthermore, PGE2 hydrogel displays obvious anti-inflammatory and pro-angiogenesis effects via inducing macrophage polarization from the M1 phenotype to M2 phenotype at injured sites. More importantly, PGE2 can reduce pathological scar formation caused by the deposition of excessive extracellular matrix (ECM) secreted by myofibroblasts because PGE2 hydrogel markedly reduces the infiltration of myofibroblasts. Besides, enhanced expression of anti-fibrotic genes and decreased collagen deposition was observed after PGE2 hydrogel treatment. Inhibiting the TGF‐β1/SMAD pathway by PGE2 might be relevant to its ability to restrain collagen synthesis in dermal fibroblasts 29. Similarly, previous data have also shown that PGE2 affects the migration and contraction of human fetal dermal fibroblasts, preventing fibrotic processes during wound healing 30, 31. In excessive wound scarring, the content of PGE2 reduces. To improve PGE2 levels, Hoon Cho et al. designed a series of 15-PGDH inhibitors 32, 33. Then, the 15-PGDH inhibitor (TD88) was investigated for its potential healing ability *in vivo* and *in vitro* 33. As a result, a significant decrease in scar formation was observed after TD88 treatment following improved reepithelization 33.

Several investigators have identified PGE2 as an angiogenesis promotor in the healing of chronic cutaneous wounds induced by diabetes diseases, in which dermal endothelial cells are seriously impaired in neovascularization, resulting in peripheral ischemia and delayed healing process 31, 34, 35. Regulating the activity of PGT to elevate the content of PGE2 in the circulation is essential for accelerating diabetes-associated wound closure 35, 36. COX-2/PGE2 pathway is increased in murine and human diabetic monocytes/macrophages. Unsuitable PGE2 activity might maintain the inflammatory phenotype of wound macrophages, which is not conducive to the repair of diabetic wounds 37. The duality of PGE2 and the complexity of the body require us to explore more to find the better therapeutic effect.

### PGE2 in myocardial injury and repair

Occlusion of the coronary artery results in myocardial ischemia that is a common clinical symptom characterized by low pH values, low oxygen, and high extracellular potassium concentration. However, myocardial reperfusion supplying oxygen and nutrients results in a series of abrupt biochemical and metabolic changes within the myocardium. Myocardial ischemia/reperfusion (I/R) injuries induce arrhythmias, myocardial stunning, microvascular dysfunction, and even myocyte death 38. Therefore, it's necessary to develop effective cardioprotective strategies and agents against myocardial I/R injury to improve myocardial function and to diminish the risk of cardiovascular events. In the heart with acute myocardial infarction, the production of PGE2 increases significantly in fibroblasts, myocardial cells, and vascular endothelial cells 39, 40. An accumulating body of evidence indicates that both exogenous and endogenous PGE2 could exert cardiac protection function against ischemia reperfusion injury 41.

Failure to effectively stimulate the proliferation of cardiomyocytes is still the main obstacle to adult heart regeneration. Cardiac stem cells can be observed not only in the infarcted area and but also in the peri-infarcted area of the injured myocardium after injury. PGE2 is an important lipid molecule that activates endogenous stem/progenitor cells for myocardium regeneration after infarction 42-44. Patrick C H Hsieh's team indicated that COX-2/PGE2/EP2 signaling promotes cardiac stem/progenitor cell differentiation into cardiomyocytes after infarction in young mice 43. Surprisingly, PGE2 also rescues the cardiomyocyte regeneration function in aged mice 43. In a study after that, Patrick C H Hsieh's team emphasized the importance of the PGE2 signaling pathway in myocardial regeneration once again 45. In zebrafish heart resection injury models, injury-induced PGE2 signaling might drive cardiomyocyte proliferation during the regeneration process 42. Moreover, by taking advantage of the natural infarct homing ability of platelet membrane and the overexpression of EP in infarcted myocardium, a novel biomimetic therapeutic nanoparticle with a platelet membrane shell conjugated with PGE2 and a core containing regeneration factors were designed to target injured heart and achieve delivery of the factors in the core. Consequently, increased cycling of cardiomyocytes, activated endogenous stem/progenitor cells, and angiogenesis was observed 44.

Both COX and mPGES-1 that determine the generation of PGE2 are required for cardiac structure repair and functional recovery following myocardial I/R injury. They are mutually irreplaceable in repair function 46, 47. There is increased cardiovascular disease risk and worse repairability, particularly in patients utilizing high-dose NSAIDs 48. Likewise, the same negative symptoms occur in animal models with genetic deletion of COX-1 or COX-2 46, 48, 49. For example, selective deletion of COX-2 in cardiomyocytes in mice can result in induced arrhythmia and reduced cardiac function. In contrast, cardiac-specific COX-2 overexpressing or constitutive expression transgene mice had small infarct size and better functional recovery in I/R injury models compared with wild type (WT) mice 41, 50. mPGES-1, the dominant synthetic enzyme for PGE2 production *in vivo*, is being considered as a new therapeutic target. Deletion of mPGES-1 in bone marrow-derived leukocytes results in impaired left ventricular (LV) remodeling such as impaired LV systolic and diastolic, leukocyte infiltration, and higher mortality after acute myocardial infarction 40. Consistently, in a myocardial infarction model, global deletion of mPGES-1can increase infarct size, reduce fractional shortening and ejection fraction 46 as well as impair microvascular perfusion via further enhancing myeloperoxidase levels and limiting leukocyte-endothelial cells interactions with EP4 receptor. These results indicate that the PGE2 signaling pathway is essential for the repair of myocardial ischemia-reperfusion and the utilization of related inhibitors such as NSAIDs should be cautious.

**Figure 2:**
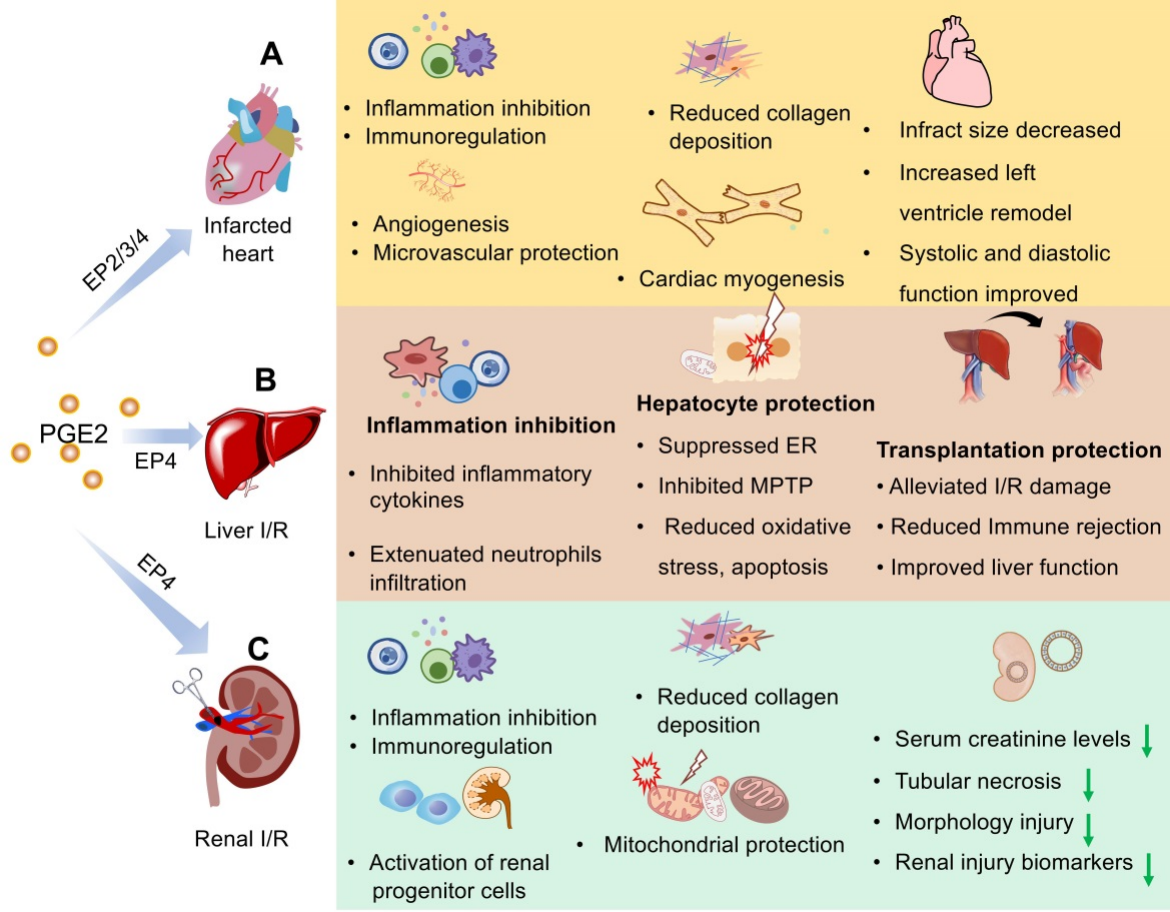
** Schematic representation of the protective effects of PGE2 in I/R injury of heart, liver, and kidney.** I/R injury that includes initial simple ischemia and subsequent reperfusion results in impaired endothelial cell barrier function, activation of cell death programs, and transcriptional reprogramming associated with inflammation and immune reaction. By binding to members of the EP family, PGE2 can decrease the inflammation response and maintain immune homeostasis in three organs with I/R injury. PGE2 can also promote angiogenesis, protect microcirculation against reoxygenation injury, and alleviate fibrosis caused by I/R injury. Apoptosis or necrosis of endothelial or epithelial cells and some parenchymal cells is prevented by PGE2. In different organs, PGE2 plays different roles. (**A**) In the myocardial infarction model, PGE2 reduces infarct size, promotes left ventricle remodeling as well as systolic and diastolic function. PGE2 also offers the benefit of cardiomyocyte regeneration. In hepatic (**B**) or renal (**C**) transplantation-induced I/R injury, PGE2 contributes substantially to the success rate and survival rate. Furthermore, PGE2 exerts remarkable effects on functional protection in organs with I/R injury. The downward green arrow indicates the inhibition effects by PGE2. ER, Endoplasmic reticulum stress; MPTP, mitochondrial permeability transition pore.

The expression levels of EP receptors may affect the role of PGE2 in the repair of myocardial I/R injury 43, 44. Among EP receptors, EP4 is the most abundantly expressed and there are more studies on EP4 in the myocardial I/R model 51-55. It's reported that overexpression of EP4 improves cardiac function after I/R injury through the reduction in adverse cardiac remodeling, inhibition of inflammation cytokine/chemokine production, and attenuated collagen deposition 51, 52 while some specific EP4 receptors agonists uncover the same protective function 51, 53, 56. As expected, cardiac function was worse after myocardial injury with various EP4 receptors and genetic deletion approaches 45, 54. Some researchers report that despite the expression of EP4 subtypes in hearts, selective stimulation of the EP2/3 receptor also displays cardio-protection against I/R injury. As we mentioned, EP2 mediates cardiomyocyte regeneration of PGE2 after myocardial ischemia via regulating macrophage activation such as migration towards the injured myocardium and phenotypic transformation from M1 to M2 in the injured myocardium 43, 45. According to previous research, cardiac specific overexpression of the EP3 receptor could mitigate ischemic contracture, creatine kinase, and lactate dehydrogenase release and improve left ventricular remodeling post- myocardial I/R injury 57. Targeted overexpression of EP3 receptors in macrophages can also facilitate cardiac healing by enhancing angiogenesis via vascular endothelial growth factor (VEGF) secretion 58. To sum up, The EP receptor is an important target for the treatment of myocardial infarction. The EP receptor agonist can mimic PGE2 to deactivate downstream signaling pathways and should be considered as a good strategy to treat MI patients (**Figure 2A**).

### PGE2 in hepatic injury and repair

Liver I/R injury is a serious complication that occurs in liver surgery, liver transplantation, and trauma. The insult induces not only damage of hepatocytes and liver sinusoidal endothelial cells but also destroys liver function and a cascade of dysfunction of other organs. Metabolic dysregulation, oxygen lack, and ATP depletion occur in hepatocytes during ischemia, leading to multifactorial hepatocyte death. Metabolically stressed hepatocytes release damage-associated molecular patterns, resulting in the induction of the innate immune response during subsequent reperfusion, while severe oxidative stress and necro apoptosis are induced because of reoxygenation. Hepatic I/R injury can be categorized into warm I/R and cold storage reperfusion injury. Warm hepatic I/R injury regularly occurs during liver resection as several procedures require intermittent total vascular occlusion and the ischemia time is relatively short. However, it's substantially longer in liver transplantation, with both cold ischemia up to 12 hours during preservation and warm ischemia during implantation, leading to increased reperfusion injury. In addition, drug-induced liver injury represents multiple reactions after exposure to any man-made or naturally occurring compound, and drug-induced hepatotoxicity is a frequent cause of liver injury 59.

Over the past 30 years, the protective role of PGE2 in liver I/R has aroused the interest of scientists 60-64. During hepatic injury, the activity of AA, PLA2, COX-2, mPGES-1, or PGE2 receptors EPs were significantly increased following I/R injury 65-68. In other words, increased hydrolysis of AA (a sort of unsaturated fatty acid) via PLA2 triggers the activity of COX and leads to increased PGE2 levels and relative downstream signals. Endogenous PGE2 is produced by many cells in the liver, mainly by hepatocytes 69, Kupffer cells 60, 61, and endothelial.

PGE2 can expedite the repair of liver ischemia-reperfusion injury by reducing liver inflammation, fibrosis, necrosis, etc. The inhibition of arachidonic acid may mediate aggravated inflammation in liver I/R injury 68. Compared with WT mice, hepatocyte-specific COX-2 transgenic mice with liver I/R injury have less hepatocyte necrosis, reduced recruitment and infiltration of neutrophils, decreased expression levels of proinflammatory factors, attenuated oxidative stress injury, and endoplasmic reticulum stress 66. It's demonstrated that COX-2 expression in hepatocytes restricts the activation of hepatic stellate cells, attenuating liver fibrosis in mice, reminding us that PGE2 may play a key role in alleviating hepatic fibrosis mediated by I/R 70. Gradually increasing PGE2 molecular secreted by activated Kupffer cells in the late period of hepatic I/R injury, it was observed to serve as a mediator to regulate and control local TNF-α release that induces inflammation reaction and organ dysregulation 60.

In response to PGE2, the role of each PGE2 receptor in liver I/R is responsible for signal transmission. All four receptors were expressed in both the naive and ischemic liver, whereas there are more experimental data about EP4 on liver I/R among all EP receptors. This may be due to injured organ-dependent or cell type-dependent. Notably, the application of EP4 agonists may contribute greatly to liver function protection in murine models of hepatic I/R injury 67, 71. It was also documented that upregulated hepatic EP4 expression could alleviate liver I/R injury by inhibiting mitochondrial permeability transition pore opening via activation of the ERK1/2-GSK3β pathway as well as ameliorating several proinflammatory cytokines, chemokines, and adhesion molecules in the early phase of reperfusion 71, 72.

However, some research results evoke an interesting paradox that deletion or inhibition of mPGES-1 or EP receptors facilitates liver repair following partial hepatic I/R injury through the accumulation of anti-inflammatory macrophages 73. In summary, there is more evidence that PGE2 is beneficial and the reverse results may be due to different ischemia reperfusion times, distinct model animals with different pathological backgrounds, or diverse treatment methods such as local or global I/R, warm or cold reperfusion.

In recent years, the role of PGE2 in liver transplantation has attracted the attention of scientists. Recipients who are measured with higher PGE2 levels in plasma may have a better graft effect 66, 74 and donors with ischemia preconditioning to elevate PGE2 levels may be protective against liver transplantation related injury 61, 66. Consistent with this, researchers making use of antibiotics for pretreatment in orthotopic liver transplantation recipients (human and mice), detected increased serum PGE2 levels and hepatic EP4 expression that suppress ER stress, enhance the autophagy pathway and improve hepatocellular function, resulting in alleviated liver transplant damage 74. PGE2 also seems to play key roles in protection against acute liver failure 75, acute graft rejection following liver transplantation 76, and other liver disorders 77.

Liver removal from a donor is also a major operation, and its trauma and complications are inevitable. Previous evidence suggests that the utilization of COX-2 antagonists accelerates liver regeneration 78, 79. Recently, researchers revealed that the liver regeneration rate and liver weight markedly increased after resection of two-thirds size in both 15-PGDH knockout mice and SW033291-treated mice compared with the control group 80. Likewise, higher levels of cyclic AMP also were observed after operation in the regenerating liver 80. Collectively, these results indicate that PGE2 has great potential in liver regeneration and is a chemical molecular promising for clinical application (**Figure 2B**).

PGE2 has also emerged as a positive regulator of drug-induced liver injury. Acetaminophen (APAP), one of the NSAIDs, and carbon tetrachloride are the two most common drugs inducing acute liver failure. In zebrafish models with APAP-induced liver injury, PGE2 has been identified as a potential therapeutic agent to decrease APAP-associated toxicity via enhancing Wnt activity 81. According to reports, sPLA2, overly secreted by the liver, is a leading mediator of the progression of hepatic injury initiated by APAP and CCl4 due to the lack of COX-2 82, 83. Furthermore, liver injury is distinctly higher in COX-2 knockout mice compared with the WT mice, emphasizing the unique contribution of PGE2 to drug-induced liver injury once again 82, 83. Celecoxib, inhibiting the activity of COX-2, aggravates liver fibrosis in CCl4-treated mice compared with the control group 84. Along with COX-2, COX-1 also protects the liver against apoptosis, oxidative stress, and inflammatory response in the CCL4-induced liver injury model 85. Lipopolysaccharide (LPS), an endotoxin composed of lipids and polysaccharides, often is utilized as an inducer of inflammation. PGE2/EP4 has been benefited to alleviate LPS-induced hepatic TNF-α expression and injury 86. In contrast, another study showed that 15-PGDH converting PGE2 to 15-keto-PGE2 may have the advantage of preventing LPS/Galactosamine induced acute liver inflammation and injury, which may be related to PPAR-γ signaling restraining inflammation 87. Taken together, these data remind us of the complexity of the body's repair mechanism and the diversified function of PGE2 under different injury backgrounds.

### PGE2 and renal I/R injury

Renal I/R injury, arising from shock or kidney transplantation, is one of the leading causes of acute kidney injury. During kidney I/R injury, initial ischemia triggers alterations in tubular endothelial structure and function, significantly leading to the overall kidney injury. The microcirculation is subsequently compromised by further vascular perfusion and oxygenation, while hypoperfusion still persists in the outer medulla of the kidney 88. There is increased vascular permeability, interstitial edema, and endothelial cell injury. Chemokines and cytokines together with other factors promote the inflammatory response leading to activation of the innate immune system as well as the adaptive immune system. If the inflammatory reaction continues within the graft tissue, progressive interstitial fibrosis will develop, whichimpacts long-term graft outcome 89, 90. Moreover, the number of peritubular capillaries is reduced after I/R injury leading to chronic hypoxia, further promoting fibrosis.

PGE2, produced by all renal cells, is the most abundant prostaglandin and plays important physiological and pathological roles in the kidney (**Figure 2C**). For instance, it participates in modulating vasopressin on osmotic water reabsorption and regulating body water metabolism 91-93. Tubular water and sodium transport, glomerular filtration, and vascular resistance are also regulated by PGE2 via its four EP receptors 91, 94, 95.

More recently, there is growing evidence regarding the pronounced effects of PGE2 on ameliorating renal I/R injury mainly via a variety of anti-oxidation, antiapoptotic, and inflammation inhibition effects. Paricalcitol treatment can prevent renal I/R with upregulated COX-2/PGE2/EP4 pathway 96, 97, reflecting the favorable role of PGE2 and EP4 in I/R injury 96, 97. EP4 agonist CAY10598 can also inhibit alterations of mitochondrial membrane potential, cytochrome C release, and cell apoptosis, as well as the energy imbalance induced by renal I/R injury 98. Excessive mitochondrial autophagy is also blocked by CAY10598 via activating the cAMP/PKA signaling pathway 98. Additionally, the expression of PGE2 may play direct or indirect roles in the immune enhancement of the damaged kidney. Previous studies reported that mesenchymal stem cells (MSCs) partially mediated Treg differentiation by the secretion of PGE2. IL-17A pretreatment could enhance the expression of COX-2/PGE2 in MSCs to increase the Treg percentage, resulting in the improved therapeutic efficacy of MSCs on renal I/R injury 99. PGE2 also exerts antifibrotic function in acute renal injury models 100. In case of the metabolism of endogenous renal PGE2, SW033291, an inhibitor of 15-hydoxyprostaglandin dehydrogenase, was administered prior to I/R injury. In addition to reduced inflammation, it was also observed that decreased injury scores, tubular apoptosis, and biomarkers of renal injury such as blood urea nitrogen, creatinine, and neutrophil gelatinase-associated lipocalin 22.

However, several data show that the synthesis of PGE2 exerts adverse effects on the repair of renal I/R. Renal fibrosis is triggered by I/R injury, which no treatment can halt or regress this process. The blockade of COX-1 and COX-2 could decrease the development of fibrosis and renal fibrosis besides their anti-inflammatory effects 101. In addition, the utilization of some COX inhibitors did reduce renal I/R injury in a dose-dependent manner 102-105. Netrin-1 suppresses the infiltration of neutrophils and macrophages by inhibiting their COX-2 expression and PGE2 production through IKB-α mediated inhibition of NF-kB in renal I/R mice 106. Moreover, it's reported that EP4 mediates the inflammatory response and ischemia reperfusion injury 106. PGE2 also reduces the expression of the proximal tubular organic anion transporters Oat1 and Oat3, exacerbating renal I/R injury 107, 108. The inconsistency between the various studies may be due to different experimental strategies such as different animal species and I/R models with different duration of ischemia or reperfusion time as well as the selection of different inhibitors.

### PGE2 in intestinal injury

PGE2 is proved that it can promote the regeneration of epithelial crypts and keep epithelial homeostasis in the face of injury induced by dextran sulfate sodium (DSS) 80, chemotherapy 109, radiation 109, colonic surgery 110, or ischemia-reperfusion 111. Both 15-PGDH knockout and 15-PGDH inhibitor (SW033291) treatment increased PGE2 levels and protected mice against DSS reduced colitis, leading to faster recovery of weight, colon length, and colitis histology scores 80. Moreover, the potential of EP4 agonists on healing and regeneration of the bowel has attracted much attention in recent years 112-114. PGE2 is conducive to wound healing of intestinal epithelial cells. A study shows that oxytocin, a drug interacting with its receptor that expresses in intestine crypt epithelial cells, also prevents intestine injury by evoking pulsatile PGE2 release 109. Under homeostasis conditions, the PGE2 signaling pathway is essential to intestinal stem cell proliferation, such as Lgr5^+^ stem cells 115, and induces stem cell differentiation towards enterocytes 116. In the face of intestinal injury, high local PGE2 levels can induce differentiation of intestinal epithelial stem cells to wound-associated epithelial (WAE) cells instead of enterocytes through EP4 and then the WAE cells migrate to cover and seal the wound bed to reestablish the epithelial barrier 116, 117.

Some signaling pathways are involved in the repair of PGE2 in intestinal injury models. YAP activity is also essential for intestinal regeneration after injury caused by DSS or radiation 118, 119. However, it is worth noting that colon cancers are highly correlated with enhanced YAP and PGE2 status 119. Another study indicated that COX-1/PGE2/EP4 also upregulates the β-arrestin1 mediated Akt signaling pathway to prevent mucosal injury in ulcerative colitis 120.

**Figure 3:**
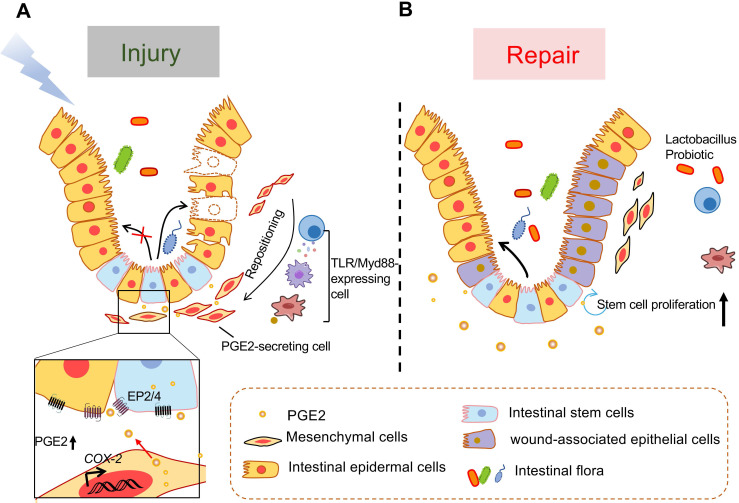
** Model of TLR/MyD88-dependent repositioning of PGE2-producing cells and of intestinal epithelial cell formation in response to mucosal injury. (A)** PGE2-secreting mesenchymal cells that mostly exist in the upper and middle portions of the rectal crypts in the steady state, migrate to the bottom of the crypt and occupy a position near the stem cell niche following intestinal injury. This migration depends on MyD88 expression by immune cells such as macrophages that are stimulated by TLR recognition of microbial products after barrier disruption. The PGE2 secreted by these newly located cells acts on intestinal stem cells or intestinal progenitor cells through EP2 or EP4 to promote their differentiation into wound-related epidermal cells. **(B)** During tissue repair, PGE2/EP4 promotes the proliferation of intestinal stem cells and the formation of intestinal epithelial cells.

The crosstalk between PGE2-secreting mesenchymal cells around the crypt and intestinal epithelial cells is critical for wound repair (**Figure 3**). In the steady state, the PGE2-producing cells include both intestinal epithelial cells and MSCs that are known as (myo)fibroblasts 121-124. MSCs are mostly present in the lamina propria of the upper and middle regions of the rectal crypt. In response to injury, they migrate to the base of the crypt to produce PGE2 depending on TLR/Myd88 expression in myeloid cells such as macrophages 115, 123, 125, 126. Both intestinal epithelial cells and lamina propria macrophages also express COX-2 in a TLR4 and MyD88-dependent manner 127. As we all know, TLRs, pattern recognition receptors expressed by immune or nonimmune cells, send signals in response to pathogen-related molecular patterns. Some substances, such as hyaluronic acid 115, 128 and Lactobacillus probiotics 122-124 have been reported that they prime the epithelial stem cell niche to protect epithelial stem cells by triggering a multicellular, adaptive immune signaling cascade involving macrophages and PGE2 secreting MSCs. Besides, exogenous adipose-derived MSCs also have a therapeutic effect on intestinal I/R injury, repairing via activating the COX-2-PGE2 signaling axis 111. It's reported that fibroblast growth factor 9 and insulin-like growth factor 2 binding protein 1 are important factors regulating COX-2 expression in colonic MSCs 121, 129.

### PGE2 and bone fracture

Bone includes the periosteum, sclerotin, and bone marrow. Among them, the periosteum is made up of fibrous connective tissue and has abundant nerves and blood vessels, which are essential for bone regeneration, sensation, and nutrition. Periosteum can be divided into inner and outer layers. Osteoclasts and osteoblasts in the inner layer of bone are responsible for the absorption and formation of bone tissue, respectively. In addition, skeletal muscle is the tissue that controls the movement of the bone. It's usually located next to the the bone and connected to bone by tendons. In response to injury, bone tissue has an extraordinary ability of self-regeneration and healing. However, large bone defects, complex fractures, or muscle damage are still major challenges facing the medical community. PGE2 is an important regulator of bone metabolism and has an anabolic effect on the repairment and regeneration of skeletal muscle tissue.

**Figure 4:**
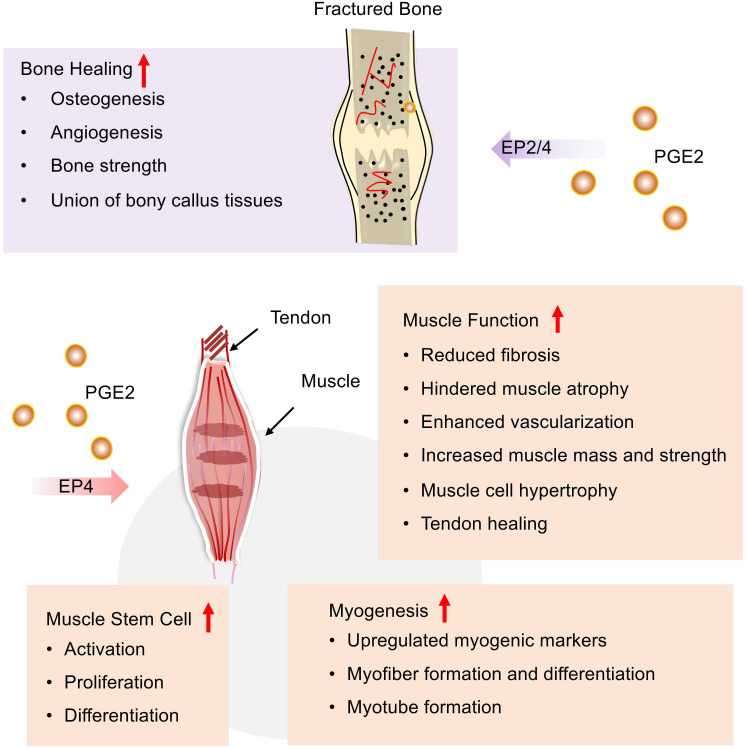
** PGE2 potentiates the repair of bone and skeletal muscle after injury.** PGE2 can not only accelerate the fracture healing process via EP2/4 but also promote the repair of muscles and tendons following injury by EP4. In the process of muscle repair, PGE2 can improve muscle function, regulate muscle-derived stem cells, as well as facilitate muscle formation.

COX-2/PGE2/EP4 signaling pathway contributes to bone fracture healing and repair (**Figure 4**). In the early inflammatory stage of fracture repair, PGE2 is mainly produced by osteoblasts 130 and found at fracture sites, while COX-2 expression regulates key subsequent events, including cartilage formation, bone formation, and remodeling. For example, PGE2 can help the new bone formation and an increase of bone mass by stimulating MSC differentiation into an osteoblastic cell line, mostly of bone marrow origin 131. Retroviral-based gene therapy with COX-2 promotes the union of bony callus tissues and accelerates fracture healing in the rat 132. The absence or inhibition of COX-2 results in impaired periosteal endochondral bone formation and marked reduction of osteogenesis and angiogenesis 133. Among all PGE2 receptors, EP4 receptors play a major role in fracture repair. Periosteal injection of EP4 agonists could markedly improve the impaired periosteal endochondral bone repair 133. EP4 agonists might help in decreasing sternal necrosis in high-risk patients or permit wider application of bilateral internal thoracic arteries in coronary artery bypass surgery, even in patients with diabetes 134. Furthermore, EP4 receptor deficiency delays fracture healing by interfering with intramembranous and cartilaginous ossification in mice 135. Additionally, an EP2 agonist has also been found to increase bone formation and strength in a rat model of femoral fracture 136. According to previous reviews, we know that COX-2/PGE2 may mediate osteoinductive communication between inflammatory macrophages and bone marrow mesenchymal stem cells, contributing to fracture healing 137.

### PGE2 and skeletal muscle

PGE2 participates in skeletal muscle repairment during all phases of healing including inflammation, regeneration, and fibrosis (**Figure 4**). COX-2/PGE2 pathway, which is mostly induced in pathological situations, is important in promoting skeletal muscle regeneration and reducing fibrosis formation 138. For example, COX-2^-/-^mice exhibits decreased skeletal muscle regeneration post-laceration injury to the tibialis anterior relative to WT mice 139. Both COX-1 and COX-2 contribute to lipid mediator synthesis production during myogenesis* in vitro*, which is most likely important for skeletal muscle myogenesis, repairment, and regeneration. In addition, AA supplementation stimulates PGE2 release and skeletal muscle cell hypertrophy via a COX-2-dependent pathway *in vitro*. Interestingly, a recent study suggests that PGE2 signaling may combat muscle atrophy and provides new insights into sarcopenia. Recently, A. R. Palla et al. revealed that elevated 15-PGDH is observed in aged tissues including skeletal muscle, leading to reduced PGE2 production. Furthermore, they uncovered that rejuvenated PGE2 levels via inhibition of 15-PGDH could augment mitochondrial function, resulting in increased muscle mass and strength 3.

Muscle-derived stem cells (MDSCs), also known as satellite cells, are crucial to muscle regeneration in response to injury 140 (**Figure 4**). PGE2 is a crucial and positive regulator of MDSCs that are known as the building blocks of muscle regeneration. PGE2 could directly target MDSCs via the EP4 receptor and results in either endogenous or transplanted MDSCs expansion, which robustly augments muscle regeneration and strength 141. Similarly, the COX-2/PGE2 signaling pathway can also stimulate transplanted MDSCs to differentiate into chondrocytes, osteoblasts, and osteocytes 142. Muscle-derived stem cell MDSCs incubated with SW033291 (15-PGDH inhibitor) can elevate the production of PGE2. *In vitro*, SW033291 enhanced myogenic differentiation and myotube formation via upregulating a series of myogenic markers with an activated PI3K/Akt pathway involved. Additionally, SW033291 incorporating the compound with MDSCs in fibrin gel significantly facilitates myofiber formation within the defect region with mild immune response, less fibrosis, and sufficient vascularization 143. Together with these findings, PGE2 can facilitate the proliferation and differentiation of MDSCs. Combined use of PGE2 and MDSC might be a nice therapy strategy to boost muscle repair after injury.

Tendons are responsible for the attachment and fixation of muscles, and the PGE2 pathway also involves the tendon healing process (**Figure 4**). In a rotator cuff tear model, Atorvastatin is known as a lipid-lowering drug, which can enhance tendon healing by stimulating tenocyte proliferation, migration, and adhesion via increased COX2/PGE2/EP4 signaling pathway 144. Post-operative adhesions forming between the tendon and the surrounding soft tissue complicate the repair process. Systemic EP4 antagonism leads to increased adhesion formation and matrix deposition, which counts with flexor tendon healing 145. Furthermore, the specific function of EP4 may be dependent on cell type 146.

### PGE2 and hematopoiesis

PGE2 regulates hematopoietic stem/progenitor cell (HSC/HSPC) homeostasis 147, 148 and modulates hematopoietic potential 149. It's known that PGE2 enhances HSC homing, engraftment, survival, and expansion 148, 150, 151. Researchers also reported that EP4 is a key receptor involving PGE2-mediated positive regulation of HSC/HSPC 152, 153. PGE2 can not only directly regulate HSC but also can stimulate the differentiation of bone marrow mesenchymal progenitor cells into hematopoietic progenitor cells via EP4 receptors in murine 152. The PGE2/Wnt interaction and cAMP/PKA signaling axis involves the regulation of PGE2 on hematopoietic stem and progenitor proliferation 78, 149.

**Figure 5:**
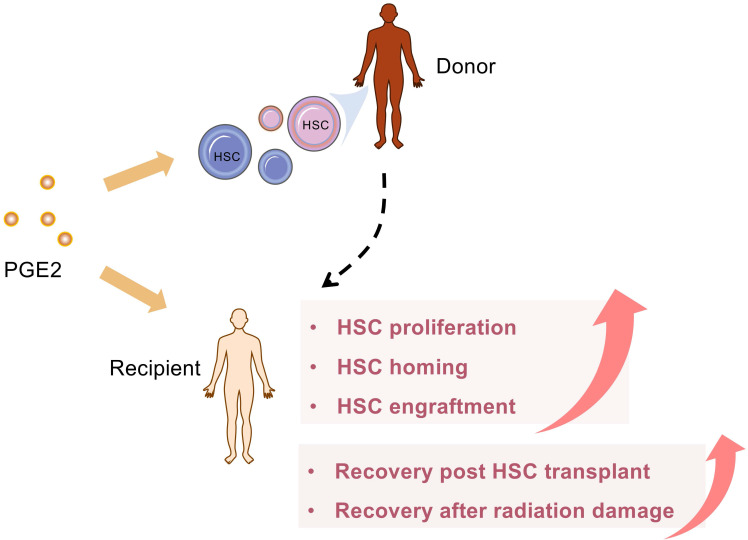
** PGE2 accelerates blood reconstitution after HSC transplantation.** PGE2 can target multiple HSC niches, therapeutically, promote hematopoietic regeneration, and improve the clinical outcome of hematopoietic stem cell transplantation. In addition, PGE2 also facilitates the homing of transplanted HSCs and accelerates the recovery from HSCs transplantation or radiation damage.

Lots of studies confirm the key role of PGE2 for hematologic reconstitution after bone marrow transplant (BMT) in detail (**Figure 5**). Importantly, 15-PGDH is becoming an attractive therapeutic target for potentiating HSC transplantation because 15-PGDH inhibitor provides a well-tolerated strategy to therapeutically target multiple HSC niches, promote hematopoietic regeneration, and improve the clinical outcomes of BMT 80, 154-156. In addition to neutrophils, 15-PGDH knockout or SW033291 treated, mice have more cell number of two specific bone marrow cell populations, which is enriched for bone marrow stem cells. Moreover, SW033291 treatment not only enhances the expression of CXCL12 and SCF in the hematopoietic niche for better supporting and homing of transplanted HSCs but also accelerates the recovery of platelets, and red cells after BMT. Consistently, SW033291 also enhances the generation of both myeloid and erythroid colonies *in vitro* 80. Subsequently, a second-generation 15PGDH inhibitor (SW209415), has been further proven to facilitate BMT recovery regardless of age, transplantation dose, or granulocyte colony stimulating factors 155. Overall, these results indicate that 15-PGDH inhibitors may have the unique advantage to increase PGE2 at physiological levels or directly at local sites, thereby avoiding extremely high concentrations caused by exogenous provision. Cautiously, overproduction of PGE2 post-BMT may impair host defense against pathogens, leading to the occurrence of infectious diseases, mainly including lung injury 157, 158. At this time, blocking PGE2 synthesis with NSAIDS or related EP receptor antagonists should be considered.

Umbilical cord blood (UCB) is a valuable source of HSC and is used for allogeneic transplantation when a suitable adult donor is not available. However, due to the low HSC content, the delay of hematopoietic and immunological recovery is still a problem to be overcome. From preclinical and clinical data, the coculture of PGE2 and cord blood stem cells is considered a cheap, safe, and practical method that can increase the homing and implantation potential of UCB stem cells 154, 159. In addition, PGE2 exhibits a strong immune regulation function. PGE2 can not only enhance the immune tolerance of UCB stem cells after transplantation but also regulate the immune reconstitution after transplantation of UCB stem cells 160, 161.

## PGE2-based therapeutic strategies for tissue regeneration

One of the limitations for using PGE2 as signal molecules is its rapid conversion under physiological conditions. As PGE2 is a key mediator of tissue regeneration and repair following injury, augmenting its effects and preventing its degradation at damaged sites are being searched as potential therapeutic strategies for a number of injured organs.

### Application of 15-PGDH inhibitors

As we mentioned earlier, PGE2 molecular is degraded into 15-keto-PGE2 in cells by 15-PGDH 19. Therefore, 15-PGDH is a negative regulator of tissue repair and regeneration in multiple organs. Scientists have been committed to the research of 15-PGDH inhibitors. Hoon Cho et al. synthesis a series of thiazolidinedione analogs 32, 162, 163 or derivatives 33, 164 to prevent the degradation of PGE2. Consequently, each of these inhibitors shows promising inhibition effects on 15-PGDH and accelerates scratch wound healing *in vitro*. However, only TD88 has been applied *in vivo* skin damage model and obtained positive results 33. It was not until the discovery of an efficient small molecule 15-PGDH inhibitor (SW033291) by Sanford D. Markowitz's team that a lot of studies were widely carried out *in vivo* 80, 165. There is no doubt that filtering such small molecule inhibitors that have a short half-life and safe pharmacological characteristics, is an outstanding job with certain application potential and clinical value.

Researchers have successively clarified the repair capabilities of SW033291 in different injury models including liver resection 80, I/R renal injury 22, intestinal injury 80, bone marrow transplantation 80, 155, 156, bone marrow failure 166, muscle defect 143, or muscle aging 3, which we have mentioned above. In fact, SW033291 was also explored in aspects of cervical ripening, drug-induced kidney injury, and pulmonary fibrosis. First, PGE2 is a cervical ripening agent, but about a quarter of full-term pregnant women who use PGE2 tablets fail to obtain a mature cervix. Furthermore, PGE2 treatment might lead to uterine hyperstimulation and abnormal fetal heart rhythms. Interestingly, a combination treatment of PGE2 and SW033291 increases the success rate of PGE2 alone-induced cervical ripening in mice 167, which should be considered in labor resulting from an unfavorable perinatal environment. Besides, since PGE2 induces vasodilation of intrarenal arteries and alleviates acute kidney injury to a certain degree, researchers also, respectively, applied SW033291 to treat contrast-induced 168 and LPS-induced 169 acute kidney injury in addition to I/R induced kidney injury 22. Consequently, SW033291 blocks intrarenal vasoconstriction as well as renal tubular cytotoxicity in contrast-induced acute kidney ischemia injury 168, while increasing the survival rate and ameliorating injury via preventing apoptosis, oxidative stress, and facilitating autophagy in LPS-induced kidney injury models 169. Additionally, a large number of reports show the protective effects of PGE2 in the bleomycin model. Studies demonstrated that suppressing PGE2 degradation with systemic administrated SW033291 shows antifibrotic effects in bleomycin-induced pulmonary fibrosis mice and human tissues 170, 171. The antifibrotic effects are specifically manifested in reduced alveolar epithelial cell apoptosis, decreased fibroblast proliferation, and diminished pulmonary fibrocyte accumulation in mice 170. Likewise, inhibitions of collagen secretion were disclosed in mice and end-stage human lung slices with bleomycin-induced fibrosis 170. Moreover, according to further investigation by Sanford D. Markowitz's team, alveolar macrophages, mast cells, as well as endothelial cells may be the key target cells 15-PGDH inhibitor therapy in murine pulmonary fibrosis models 171.

On the one hand, even though SW033291 has high 15PGDH inhibition efficiency and superior repair-promoting function, it still requires further optimization to adapt to different injury backgrounds without altering the potency inhibiting 15PGDH. For instance, it's better to utilize intravenous administration drugs with high aqueous solubility such as in hematopoietic stem cell transplantation patients. Sanford D. Markowitz's team developed a second-generation 15-PGDH inhibitor, SW209415, with 10,000-fold more solubility compared with SW033291 155, facilitating recovery from hematopoietic stem cell transplantation. On the other hand, the application of 15-PGDH inhibitors is not just limited to regenerative medicine. For example, a study reports that reduced PGE2 levels in patients lead to anaphylaxis via resulting in mast cell hyperresponsiveness. Then the 15-PGDH inhibitor elevating PGE2 levels decreases the severity of the disease in murine models with passive systemic anaphylaxis 12. Of note, the utilization of 15-PGDH inhibitors might be positively correlated with the risk of cancer and maybe should not be considered in this special context 172-174.

### PGE2 delivery systems

**Figure 6:**
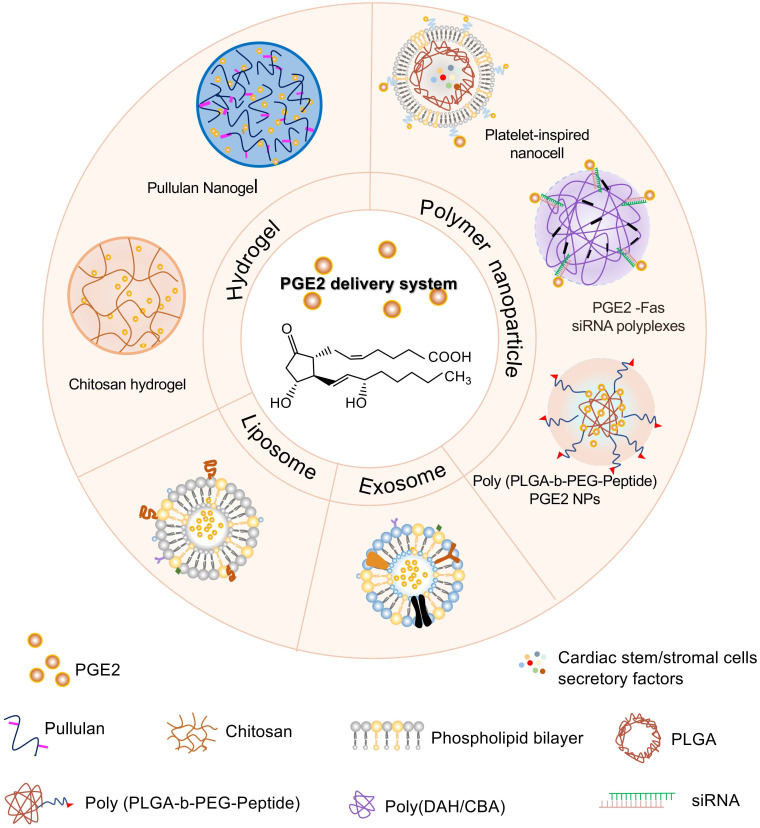
** Different PGE2 biomaterial delivery systems.** PGE2 delivery system is mainly divided into three categories including injectable hydrogels, liposomes or exosomes, and polymeric nanoparticles. Different hydrogel scaffolds containing PGE2 such as chitosan and pullulan hydrogel, contribute to the sustained release of PGE2. Exosomes and liposomes formed by the phospholipid bilayers encapsulate PGE2 within the hydrophilic core. Polymeric nanoparticles loading PGE2 are composed of natural or synthetic polymers such as poly (DAH/CBA) and PLGA. Platelet-inspired nanocell consists of an outer platelet-derived phospholipid bilayer binding PGE2 and inner PLGA nanoparticles encapsulated with cardiac stem/stromal cell secretory factors. PGE2 conjugation with siRNA to silence the apoptosis-related Fas genes is condensed with a reducible degradable cationic copolymer (poly (DAH/CBA)). Consistent with the platelet-derived nanocells, PGE2 molecules are exposed on the surface of the copolymer for targeting. The self-assembly of PLGA-b-PEG copolymer linked to an endothelial-targeted peptide forms poly PLGA-PEG-Peptide polymer encapsulating PGE2 in the core. PLGA, poly lactic-co-glycolic acid; poly (DAH/CBA), poly (1,6-diaminohexane, and cystamine bisacrylamide); PLGA-b-PEG, a poly (lactide-coglycolide acid)-b-poly (ethylene glycol) (PLGA-b-PEG) copolymer.

How to deliver exogenous PGE2 to the sites of action is worthy of exploration besides increasing endogenous PGE2 levels. Direct administration of PGE2 seems not to be ideal because it has a very rapid turnover rate *in vivo* and is eliminated from tissues or circulation even in some successful cases. Currently, several biological materials delivery systems have already been designed and synthesized to control PGE2 release, which increases the treatment efficacy of PGE2 and has potential applications in regeneration medicine. Those delivery vehicles include injectable hydrogels, liposomes or extracellular vesicles, and polymeric nanoparticles (**Figure 6**).

#### Injectable hydrogel

Hydrogel is a cross-linked network of hydrophilic polymers, capable of holding plenty of water but remaining insoluble and keeping their three-dimensional structure 23, 175. In recent years, injectable hydrogel has raised increasing attention to their application as reservoir systems for effector molecule delivery so that biodegradability and biosorption can be ensured after appropriate design 4, 176, 177. Pharmacokinetic studies have also revealed that effector molecules encapsulated within the hydrogel matrix could prolong the life of PGE2 in the circulation or wound surrounding tissues 178-180.

The application of the PGE2 sustained-release system may originate from Prepidil (an intracervical PGE2 gel) and Cervidil (a controlled-release hydrogel pessary) used as a cervical ripening agent 181. Our previous study revealed that incorporating PGE2 with chitosan hydrogel to prolong the release of PGE2 contributes to improved cutaneous repair and regeneration 2. PGE2 hydrogel significantly improves wound healing and ameliorates inflammation by promoting the polarization of M2 macrophages. Moreover, several nanogels based on cholesterol-bearing pullulan polysaccharides (CHP) encapsulating PGE2 exert positive effects on enhanced bone formation 182, while free PGE2 do not increase the thickness of the calvariae. Similarly, a biodegradable temperature-sensitive hydrogel permitting controlled release of PGE2 also plays beneficial effects on post-infarction ventricular function via preserving the immune privilege of implanted allogeneic MSCs that contribute to cardiac ischemia reperfusion repairment 183.

#### Liposomes or extracellular vesicles

Liposomes are spherical lipid bilayer vesicles formulated with an outer lipid bilayer that consists of natural, synthetic, or modified lipid species and an internal aqueous core 184. Therefore, these structures can carry hydrophilic molecules in the core and hydrophobic ones between the bilayers. The lipophilic character of the liposomes makes it possible to be an efficient delivery system to protect the drugs against chemical or enzymatic degradation. T. Minko et al. demonstrated that *ex vivo* liposome containing PGE2 delivered locally to the pulmonary by inhalation was an effective therapeutic strategy to prevent idiopathic, pulmonary fibrosis through limiting fibroblast proliferation, activation, migration, collagen secretion, and/or myofibroblast differentiation 185. Furthermore, extracellular vehicles (EVs) also have a phospholipid bilayer structure, which act as intermediaries between the membrane and cytoplasmic proteins, lipids, and RNA 24, 186, 187. It's known that most EVs will accumulate in the liver after intravenous injection 188. Moreover, a study showed that EVs-like nanoparticles loading PGE2 that derive from the intestine mucus could be transferred to the liver to maintain liver NK T cell homeostasis 189. Therefore, EVs carrying PGE2 have potentially valuable therapeutic capabilities for a series of liver injuries. Another possibility is that engineering EVs with targeting functions may deliver PGE2 to the injured sites accurately 190. PGE2 stored in liposomes or EVs might not directly act on EP receptors at the intracellular lipid membrane surface because of endocytosis. In conclusion, EVs, as a next-generation drug delivery platform, have extensive application prospects 191.

#### Polymeric nanoparticles

Polymeric nanoparticles are composed of many materials and components, mainly including natural or synthetic polymers so that satisfying characteristics such as good biocompatibility, broad structure variety, and noticeable bio-imitative are available. Polymeric nanoparticles can transport the drug to targeted tissues or organs and boost delivery efficiency via adjusting the chemical or physical characteristics of the polymer matrix 192, 193.

As we have known, EP receptors have a higher level of expression in myocardial cells after myocardial infarction, and the apoptosis-related genes, Fas and Fas ligands, are overexpressed in apoptotic cardiomyocytes. PGE2-Fas siRNA synthesized via the conjugation between PGE2 and Fas siRNA molecules. Subsequently, PGE2-siRNA conjugation is tightly condensed with reducible and degradable cationic copolymers synthesized by Michael-type polyaddition of 1,6-diaminohexane and cystamine bisacrylamide (poly (DAH/CBA)) to form nanosize polyplexes. This kind of polymeric nanoparticles is intracellularly degraded and releases siRNA after PGE2 receptor-mediated endocytosis, which inhibits cardiomyocyte apoptosis significantly after myocardial infarction 194. Recently, scientists designed a platelet-inspired nano cell (PINC) consisting of a core and a platelet membrane shell conjugated with PGE2. The core is composed of resident cardiac stem/stromal cell secretome-loaded PLGA nanoparticles. Experimental results proved that the PINC linking PGE2 increases cycling cardiomyocytes, activates endogenous stem/progenitor cells, and promotes functional recovery more effectively compared to the groups without PGE2, which makes use of the dual features of PGE2 including targeting to cardiovascular cells and facilitating endogenous repair 44.

For PGE2 stimulated angiogenesis, Robert Langer *et al.* utilized a poly (lactide-coglycolide acid) - b - poly (ethylene glycol) (PLGA-b-PEG) copolymer to encapsulate 16,16-dimethyl PGE2 (a stable PGE2 analog) or another angiogenic stimulator to form vasculature-targeted polypeptide-nanoparticle, providing us with new avenues in exploring the therapeutic potential of PGE2 195.

### The future of PGE2-based treatment strategies

**Figure 7:**
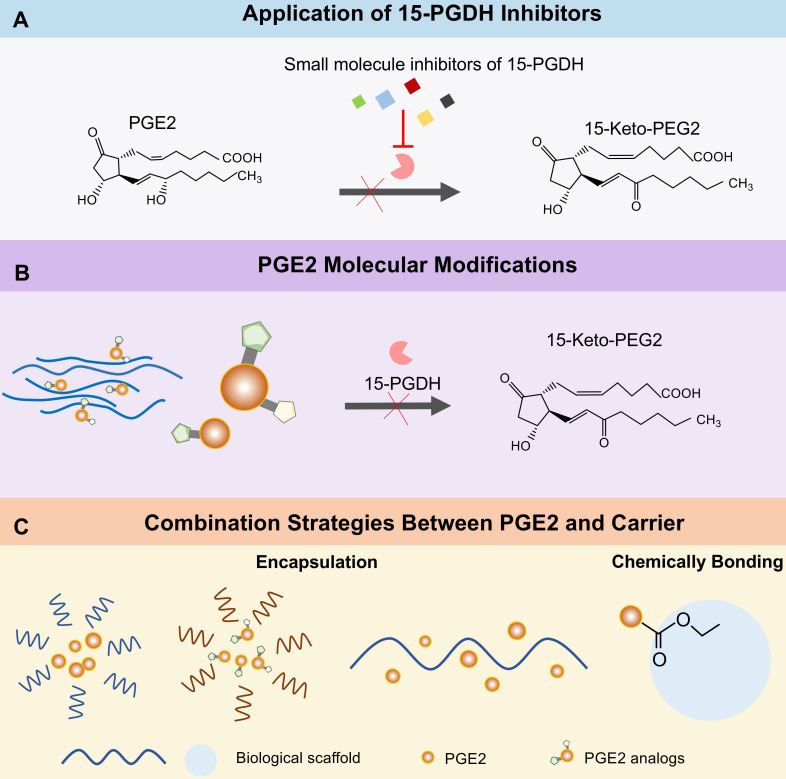
** Therapeutic strategies for enhancing tissue regeneration of PGE2. (A)** Inhibiting the activity via several small molecular compounds of 15-PGDH prevents PGE2 from degradation.** (B)** Modifying the PGE2 molecule to change its spatial structure or dehydrogenation site can resist the metabolism of 15-PGDH without changing the activity of PGE2. **(C)** Strategies for controlling release of PGE2 from biomaterials.

In recent years, 15-PGDH inhibitors have acquired convincing data in extensive basic research and revealed potential in clinical regeneration therapy. Intracellular PGE2 metabolized by 15-PGDH leads to the suppression of the downstream signals, which can be reversed by 15-PGDH inhibitors (**Figure 7A**). The development of 15-PGDH inhibitors with different mechanisms of action and stable drug characteristics will be able to provide different treatment options to patients with different types of injuries. Rodeo Therapeutics Corporation focuses on the exploration of small molecule therapies to promote regeneration and repair of diverse tissues. Its first oral 15-PGDH modulator, RTX-1688, was designed to treat intestinal diseases and this preclinical project has broad application potential. In addition, an alternative strategy to enhance the self-stability of PGE2 by chemical modification 195, such as adding or replacing some functional groups, is also promising for PGE2 based therapy. PGE2 analogs with spatial structures different from PGE2, are not subordinated to the degradation of 15-PGDH **(Figure 7B)**. For example, previous experiments have discovered that 15-methyl PGE2, or 16, 16-dimethyl PGE2 are more stable than PGE2 when administered intravenously, orally and intrajejunal 196-198. Besides, PGE2 analogs have been widely used in clinical practice for cervical ripening and labor induction 199. Another strategy available is the incorporation of engineered biomatrices for PGE2 control release by different approaches, such as encapsulation and chemical bonding (**Figure 7C**). The limitations of current polymer drug delivery systems indicate that once drug molecules are encapsulated, they are susceptible to diffuse out of the nanocarrier. Hence, chemically bonding PGE2 to multifunctional biomaterials through stimulation-sensitive linkers, it may open new vistas in the design of smart and controllable release systems. How to accurately locate PGE2 delivery materials to damaged organs/sites in the body is a key issue that needs to be overcome, which is highly demanded for the development of optimized PGE2 delivery systems.

## Conclusions and future perspectives

A broad range of studies *in vitro* and *in vivo* suggests that the PGE2 signaling pathway could protect different organs against injury from inflammation, oxidative stress, or fibrosis. Collectively, prior and current studies have been exploring efficient 15-PGDH inhibitors and have confirmed SW033291 as an ideal one that potentiates repair and regeneration of diverse organ systems following injury with increased PGE2 production. In addition, biomaterial delivery strategies have been proven effective, which reminds us that we should pay close attention to the potential combination therapies between PGE2 and the delivery system. In summary, the content presented in this review not only provides the mechanisms by which PGE2 works against injury in different organs, but also investigates the application of PGE2-based therapeutic strategies. Extensive exploration is necessary to further uncover the therapeutic potential of PGE2 in regeneration areas.

## Abbreviations

AA: arachidonic acid
AC: adenylate cyclase
APAP: acetaminophen
ATP: adenosine triphosphate
cAMP: cyclic adenosine monophosphate
BMT: bone marrow transplant
CHP: cholesterol-bearing pullulan polysaccharides
CD206: cluster of differentiation 206
COX: cyclooxygenase
DAH/CBA: 1,6-diaminohexane/cystamine bisacrylamide
DSS: dextran sulfate sodium
ECM: extracellular matrix
EP: E-type prostanoid
ER: endoplasmic reticulum
ERK1/2: extracellular signal-regulated kinase1/2
EVs: extracellular vehicles
GSK3β: glycogen synthase kinase 3β
HSC/HSPC: hematopoietic stem/progenitor cell
I/R: ischemia/reperfusion
LPS: lipopolysaccharide
LV: left ventricle
MDSCs: muscle-derived stem cells
MI/R: myocardial ischemia reperfusion
MRP4: multidrug resistance protein 4
MSCs: mesenchymal stem cells
NSAIDs: nonsteroidal anti-inflammatory drugs
15-PGDH: 15-hydroxyprostaglandin dehydrogenase
PGE2: prostaglandin E2
PGES: prostaglandin E synthase
cPGES: cytosolic prostaglandin E synthases
mPGES-1/2: microsomal prostaglandin E synthases1/2
PGH2: prostaglandin H2
PGS: prostaglandin synthase
PGT: prostaglandin transporter
PI3K: phosphoinositide 3-kinases
PKA: protein kinase A
PLA2: phospholipase A2
sPLA2: secretory phospholipase A2
PINC: platelet-inspired nano cell
PLGA-b-PEG: poly (lactide-coglycolide acid) - b - poly (ethylene glycol)
PPAR- γ: peroxisome proliferators-activated receptors-γ
RELM-α: resistin-like molecule-α
SLCO2A1: solute carrier organic anion transporter family member 2A1
SMAD: drosophila mothers against decapentaplegic protein
TGF-β: transforming growth factor-β
TLR: toll-like receptor
TNF-α: tumor necrosis factor-α
UCB: umbilical cord blood
VEGF: vascular endothelial growth factor
WAE: wound-associated epithelial
WT: wild type
